# Amphisbaenians facultatively oviposit in ant and termite nests

**DOI:** 10.1007/s00114-026-02132-0

**Published:** 2026-07-16

**Authors:** Henrique Bartolomeu Braz, Lívia Cristina Santos, Selma Maria Almeida-Santos

**Affiliations:** 1 Laboratório de Ecologia e Evolução, Instituto Butantan, Av. Vital Brazil, 1500, São Paulo, SP 05503-900 Brazil; 2 Instituto Federal de São Paulo, Av. Prof. Celso Ferreira da Silva,1333, Avaré, SP 18707-150 Brazil; 3 Faculdade de Medicina Veterinária e Zootecnia, Universidade de São Paulo, Av. Prof. Orlando Marques de Paiva, 87, São Paulo, SP 05508-270 Brazil

**Keywords:** Oviposition-site choice, Maternal decision-making, Behavioral plasticity, Fossorial reptiles

## Abstract

Oviposition-site selection is a key maternal decision in oviparous animals, involving trade-offs among incubation conditions, offspring performance, and maternal constraints. In amphisbaenians (worm lizards), a clade of highly specialized fossorial reptiles, oviposition has long been assumed to occur obligatorily in ant and termite nests, implying strong ecological specialization. Here, we re-evaluate this assumption using new field observations and a critical synthesis of published records. We describe a natural oviposition site of the smallhead worm lizard (*Leposternon microcephalum*) located in a soil cavity with no evidence of ants or termites and review 18 records from nine amphisbaenian species. Across species, eggs occur not only in ant and termite nests but also in subterranean cavities and decaying logs, indicating facultative rather than exclusive use of social-insect nests. A field inspection of 31 active ant nests during the reported oviposition season yielded no eggs or amphisbaenians. Additional evidence suggests some species excavate or modify underground chambers and that at least one species oviposits communally. These findings challenge the view of strict dependence on ant and termite nests and instead support oviposition-site choice in amphisbaenians as a flexible, context-dependent maternal behavior shaped by ecological trade-offs rather than rigid specialization. Standardized surveys across microhabitats and experimental tests of incubation environments are needed to clarify how availability, costs, and benefits interact to shape nesting decisions in fossorial reptiles.

## Significance statement

How animals choose where to lay their eggs can strongly influence offspring survival, yet this decision remains poorly understood in secretive, underground species. Worm lizards (amphisbaenians) have long been thought to depend on ant and termite nests for egg-laying, implying rigid ecological specialization. By combining new field observations with a critical reassessment of published records, this study shows that such behavioral decision is not obligatory. Instead, amphisbaenians use social-insect nests as part of a broader and flexible set of nesting options. Our findings highlight oviposition-site choice in these reptiles as a context-dependent maternal decision shaped by trade-offs among environmental conditions, risks, and constraints, rather than fixed dependence on a single nesting strategy. This work reframes amphisbaenian reproduction within a behavioral decision-making framework and underscores the importance of considering plasticity and detectability biases when interpreting nesting ecology in fossorial animals.

## Introduction

*“The reason for this association has not yet been determined. Brazilians hold the pious belief that ants take pity on the blind lizard and welcome it into their nests*,* even bringing it food!”* (*Tschudi 1866: 159*).

For oviparous animals in which embryos develop outside the maternal body, oviposition-site choice is a major determinant of reproductive success. In many of these taxa, parental care is limited or absent, so oviposition-site choice becomes a major maternal effect through which mothers influence offspring survival and phenotype (Resetarits 1996; Refsnider and Janzen 2010). The physical and biological attributes of the chosen site influence embryonic development and hatching success by modulating temperature, humidity, gas exchange, microbial activity, and predation risk (Refsnider 2016; Buxton and Sperry 2017; Schütz and Füreder 2019; Fowler et al. 2025). The relevance of these factors, however, varies widely among taxa and depends on species-specific life-history traits, creating trade-offs among maternal survival, incubation conditions, and offspring performance (Refsnider and Janzen 2010). Thus, oviposition-site selection is best viewed as a maternal reproductive decision with direct fitness consequences.

In reptiles, temperature and moisture at the oviposition site can influence embryonic growth, hatching success, and a range of offspring traits, sometimes with long-term effects. Site structure and local biota also affect egg survival by altering predation risk, microbial colonization, and exposure to flooding or desiccation (Refsnider 2016; Abayarathna and Webb 2022). Together, these pressures have produced a wide diversity of oviposition strategies that differ in the degree of site preparation, from the use of unmodified, pre-existing shelters (e.g., rock crevices, decaying logs, or colonies of social insects) to actively excavated burrows or constructed nesting structures (Packard and Packard 1988; Refsnider 2016; Murray et al. 2020). Variation also occurs in how sites are used. Females may oviposit alone (solitary oviposition) or share sites with other females (communal oviposition). Communal oviposition is often associated with limited availability of suitable sites or with shared benefits such as stable microclimates or reduced predation risk (Graves and Duvall 1995; Doody et al. 2009). Despite the relevance of these maternal decisions, current knowledge of reptile nesting ecology and behavior is strongly biased toward large, surface-active taxa (Doody et al. 2021; Doody and Refsnider 2022), leaving fossorial, limbless, and secretive lineages underrepresented in comparative studies.

This bias is especially pronounced in amphisbaenians (worm lizards), a monophyletic and highly specialized group of ~ 200 species of fossorial, mostly limbless reptiles (except for the forelimbed *Bipes*), distributed across tropical and subtropical regions of the Americas, Africa, and parts of the Mediterranean and Middle East (Pough et al. 2003; Uetz et al. 2025). Their subterranean lifestyle severely limits direct field observations; consequently, fundamental aspects of their nesting ecology and nesting-related behavior are poorly documented, hindering the identification of general patterns (Andrade et al. 2006). This lack of information can also generate detection bias. Conspicuous microhabitats are more likely to be searched and reported, which can inflate the apparent importance of particular oviposition sites. The same limitation can bias inferences about how sites are used. For instance, communal oviposition has not been documented in amphisbaenians; however, given how widespread this behavior is among other squamates (Graves and Duvall 1995; Doody et al. 2009), its apparent absence in amphisbaenians may reflect limited observations rather than a true lack of communal nesting.

From a functional perspective, it has been assumed that limblessness constrains nest construction, such that limbless reptiles, including amphisbaenians, rely mainly on pre-existing structures (e.g., decaying logs, rock crevices, insect colonies) for oviposition rather than constructing nests (Packard and Packard 1988; Doody et al. 2009; but see Burger and Zappalorti 1991; Koirala and Tshering 2021 for exceptions). Riley et al. (1985) reviewed records of squamate oviposition in ant and termite nests and noted that eggs of amphisbaenians (*Amphisbaena alba*, *A. kingii*, and *Leposternon microcephalum*) had been reported exclusively from ant nests. They interpreted this pattern as consistent with a possible obligate association (i.e., exclusive or near-exclusive use) with ant nests for oviposition. However, for amphisbaenians, this inference may not reflect a true functional constraint. As specialized burrowers, amphisbaenians possess morphological and behavioral traits that could allow them to excavate or modify oviposition chambers in the soil, rather than relying solely on pre-existing shelters. This “obligate ant-nest” hypothesis, although cited by later authors (e.g., Azevedo-Ramos and Moutinho 1994; Vega 2001; Andrade et al. 2006), has never been explicitly assessed, largely because natural oviposition sites are rarely documented for this group. Given the many records of amphisbaenians occurring in termite nests (Broadley et al. 1976; Vega 2001; Pramuk and Alamillo 2003; Moreira et al. 2009; Duleba and Ferreira 2014), and because various other limbless squamates also oviposit in such sites (Riley et al. 1985), this idea can reasonably be extended to termite nests as well (FitzSimons 1943; Bons and Saint Girons 1963). Proposed benefits of ovipositing in ant and termite nests include stable microclimates, protection from predators or microbes, and immediate access to food resources (Hagmann 1907; Kopstein 1928; Vaz-Ferreira et al. 1970; Riley et al. 1985; Hood et al. 2020). If amphisbaenians truly depend on ant and termite nests as exclusive or near-exclusive oviposition sites, this would imply a high degree of ecological and behavioral specialization, potentially involving coevolutionary mechanisms such as insect tolerance toward eggs or traits that facilitate persistence within active colonies (Baer et al. 2009; Sierra-Serrano et al. 2023). Testing whether such dependence exists is therefore necessary to distinguish specialization from flexibility in a key reproductive behavior.

Here, we evaluate the hypothesis that amphisbaenians obligatorily oviposit in ant and termite nests. First, we describe the rare finding of a natural oviposition site of *L. microcephalum*—a species previously suggested to oviposit exclusively in ant nests (Riley et al. 1985)—found in a soil cavity with no evidence of ants or termites. We then compile and critically review published records of amphisbaenian oviposition sites to assess whether ant and termite nests are obligatory for oviposition and whether these sites are used solitarily or communally. If oviposition in ant and termite nests were obligatory, eggs should be restricted to these sites; in contrast, facultative use predicts oviposition across diverse microhabitats. Finally, we discuss the potential costs and benefits that may drive the use of ant and termite nests in amphisbaenians.

## Materials and methods

### Original data

On 26 February 2011, three eggs were found in a private garden in Praia Grande municipality, São Paulo state, southeastern Brazil (24° 00’ 21.6” S, 46° 24’ 10.8” W; elevation: 11 m) during routine maintenance. The eggs were collected, placed in a plastic bag with soil from the same site, and transported to the laboratory the following day. Two eggs hatched during transportation. The remaining egg was measured with a digital caliper (to the nearest 0.01 mm), weighed (to the nearest 0.01 g), returned to the bag, and kept at room temperature. Hatchlings were measured (to the nearest 1 mm), weighed (to the nearest 0.01 g), photographed, and deposited in the Reference Collection of the Butantan Institute (IBSP.CRIB) and Museu de Zoologia da Universidade de São Paulo (MZUSP).

On 15 December 2007, we manually opened 30 leaf-cutting ant (*Acromyrmex*) nests in a *Hevea brasiliensis* plantation and one *Atta* nest in adjacent pasture areas (ca. 1 ha) at Fazenda Novo Mundo (22° 19′ 45″ S, 49° 45′ 23″ W), Vera Cruz municipality, São Paulo state, southeastern Brazil. Colonies of *Acromyrmex* are the ant nests most frequently reported as oviposition sites for squamates (Sacerdote-Velat and Sekits 2023). For each nest, we opened chambers as far as feasible and visually searched for eggs or amphisbaenians within the nest structure and adjacent soil. During the survey, we also searched beneath logs and rocks encountered within the sampled area for amphisbaenians, eggs, or nests. The surveyed area harbors multiple amphisbaenian species (Mott and Vieites 2009; Santos 2013), and December coincides with the oviposition period reported for amphisbaenians in southeastern Brazil (Santos 2013; see also references in Table 1).

**Table 1:** Summary of available records on natural oviposition sites of Amphisbaenia. The column ‘Information originally provided’ reproduces the original account as closely as possible, while the columns ‘Nest site’ and ‘Nest use’ represent our standardized interpretation. NA = not available

Species	Location	Information originally provided	Nest site	Nest use	Reference
Unidentified amphisbaenid ^a^	Rio de Janeiro, Brazil	Eggs are laid within fungus chambers of old nests of leaf-cutting (“*tanajura*”) ants.	Ant nest (fungus chamber, Formicidae, Myrmicinae, Attini)	NA	Tschudi 1867; Brandão and Vanzolini 1985
*Amphisbaena caeca*	Puerto Rico	One egg found beneath a termite nest.	Below termite nest	Solitary	Schmidt 1920
*Amphisbaena caeca*	Puerto Rico	Two eggs and one adult specimen found ~ 7.5 cm under a log beneath an ant’s nest on 22 August 1919. One egg (42 × 11 mm) contained a hatchling-sized specimen (86 mm total length) and residual yolk mass (AMNH 13237).	Under log	Solitary	Schmidt 1920; Gans and Alexander 1962
*Amphisbaena darwinii*	Uruguay	Eggs found in layers of humus.	Underground (humus layer)	NA	Vaz-Ferreira et al. 1970
*Amphisbaena darwinii*	Uruguay	A clutch of four eggs (mean egg size = 24.7 × 13.0 mm) found within an unoccupied anthill (species unidentified) in a grassland habitat on 30 December 2002. Three eggs hatched on 25–26 January 2003 (hatchling total length = 68, 74, and 85 mm; ZVC-R 6080).	Ant mound	Solitary	Carreira and Baletta 2006
*Amphisbaena darwinii*	Argentina	A female and a clutch (egg length ~ 20 mm) of two eggs containing near-term embryos were found in a small cavity in the soil dug from a garden in February 1895.	Underground cavity	Solitary	Berg 1898
*Amphisbaena darwinii*	Argentina	A female and a clutch of three eggs (egg length ~ 20 mm) containing near-term embryos were found in a small cavity in the soil dug from a garden in February 1896.	Underground cavity	Solitary	Berg 1898
*Amphisbaena darwinii*	Argentina	Eggs (clutches ranging 6–8 and 17–21) found under logs of an old *Eucalyptus* crop partially buried in loose soil, which contained two or more species of termites and ants. Eggs and embryonic series housed at UNNEC (see Montero et al. 1999).	Under log in loose soil (termite and ant presence)	Solitary and communal ^b^	Montero et al. 1999; J. A. Céspedez, pers. comm.
*Amphisbaena fuliginosa* ^c^	Brazil	A clutch of nine eggs (one egg measuring 28.22 × 14.74 mm) was found on 2 October 2016, partially buried beneath a fallen tree trunk. Eggs hatched on 19–20 October (hatchling size = 122–135 mm total length). An adult female *A. fuliginosa* was also found in the nest site.	Under log	Solitary	Oliveira and Gomes 2017
*Amphisbaena kingii*	Argentina	One egg (29 × 11 mm) containing a fully developed embryo (75 mm total length) was found in early March 1988 at the base of the fungus chamber of a nest of *Acromyrmex silvestrii* (MLP S.1097).	Ant nest (fungus chamber, Myrmicinae, Attini)	Solitary	Williams and Wichmann 1989
*Amphisbaena kingii*	Brazil	Several (≥ 2) eggs found in ant nests. Two of these eggs were examined by Gans and Rhodes (1964); the eggs (30 × 10 and 35 × 10 mm) contained fully developed embryos (113 mm total length; NHMUK 1885.2.3.6-7).	Ant nest	Probably solitary	Boulenger 1885; Gans and Rhodes 1964
*Amphisbaena mertensii*	Paraguay	A gravid female (301 mm SVL; MNHNP 8742) with stomach contents (Coleoptera, Elateridae) was found buried in the softer soil peripheral to a termite (*Cornitermes cumulans*) mound; no eggs recorded.	Termite nest (peripheral soil)	NA	Pramuk and Alamillo 2003
*Leposternon infraorbitale*	Brazil	A clutch of six eggs (egg size = 60 × 25 mm) found on 20 February 1994 in the ground beside a fallen tree in a cacao plantation, 15 cm from the surface and covered in soil and decomposed wood. Hatchlings 150–170 mm total length.	Under decomposing wood	Solitary	Jared et al. 1997
*Leposternon microcephalum*	São Paulo, Brazil	A clutch of three eggs (one egg measuring 45.26 × 15.31 mm) found in a cavity 18 cm below the soil surface in a small garden located inside a private residence on 26 February 2011. Two eggs hatched the next day. Hatchlings measured 126, 136, and 142 mm in total length (IBSP.CRIB 295 and 296; MZUSP 103179).	Underground	Solitary	Present study
*Leposternon microcephalum* ^d^	Brazil	Eggs (unreported number) were taken in March 1893 from a pile of bricks and fragments of roof tiles inhabited by a reasonable size colony of ants (*Camponotus*). Two sets of eggs were found: one with less-developed embryos, another with embryos close to hatching. Two eggs of the older clutch measured 53.5 × 20 and 54.5 × 18 mm. Two other eggs (52 × 12 and 45 × 14 mm) are vouchered as NHMUK 1893.9.30.2–3.	Ant nest (Formicinae, Camponotini)	Possibly communal	Goeldi 1898
*Leposternon microcephalum*	Brazil	A clutch of two eggs was found in an oval (8.0 × 3.5 × 4.5 cm) smooth-walled cavity 23–25 cm below the surface of a truck garden. The soil was sandy-loamy interspersed with hummus.	Underground cavity	Solitary	Engmann 1927
*Leposternon microcephalum* ^e^	Brazil	Two eggs (ZMH 3571) containing partially developed embryos (39 mm total length) collected in an anthill on 17 December 1907.	Ant mound	Solitary	Gans 1971
*Leposternon microcephalum* ^e^	Brazil	Eight eggs (ZMH 3572) containing early embryos collected in an anthill in the spring of 1908.	Ant mound	Possibly communal (due to the clutch size)	Gans 1971
*Rhineura floridana*	Florida, United States	A clutch of two eggs (one egg measuring 38.0 × 8.9 mm) containing fully formed young (93.5 and 112.2 mm in total length) was found after turning up in a spadeful of sandy loam taken from a depth of 20–50 cm.	Underground (sandy soil)	Solitary	Carr 1949

^a^ Tschudi (1867) reports *A. flavescens* and *A. fuliginosa* inhabiting “tanajura” nests and laying eggs there; however, the original text is ambiguous as to which species (or whether both) oviposit in such sites. *Amphisbaena flavescens* is now recognized as *A. alba*, whereas *A. fuliginosa* does not occur in the region. Moreover, five other amphisbaenian species are currently known from Rio de Janeiro state (Guedes et al. (2023). Given this uncertainty, we list the record as “unidentified amphisbaenid”

^b^ Given that *A. darwinii* typically lays 2–4 eggs (Gallardo 1967; Carreira and Baletta 2006), these aggregations likely represent oviposition by multiple females (~ 2–11)

^c^ Originally published as *A. brasiliana* but identified as *A. fuliginosa* by Abecassis et al. (2026)

^d^ Gans (1971) comments that “it is uncertain that the eggs belong to species *microcephalum*”

^e^ Identified as *L. microcephalum* by Gans (1971)

### Literature survey

To assess the hypothesis of obligatory oviposition in ant or termite nests in amphisbaenians, we searched the literature for records of natural oviposition sites. We searched Google Scholar using combinations of “Amphisbaenia”, “amphisbaenian”, “worm lizard”, genus names, and keywords such as “nest”, “oviposition”, “egg-laying”, and “egg”, in Portuguese, Spanish, and English. We extracted descriptions of oviposition microhabitats and classified nesting as solitary or communal when possible. To distinguish between solitary and communal nesting, we used multiple criteria, including developmental stages of embryos within nests, the time interval between hatching events, and whether the number of eggs at a site exceeded the mean clutch size reported for the species (Braz et al. 2008). We also compiled clutch size, egg size, offspring size, and incubation duration when available.

## Results

### Original data

**Fig. 1:**
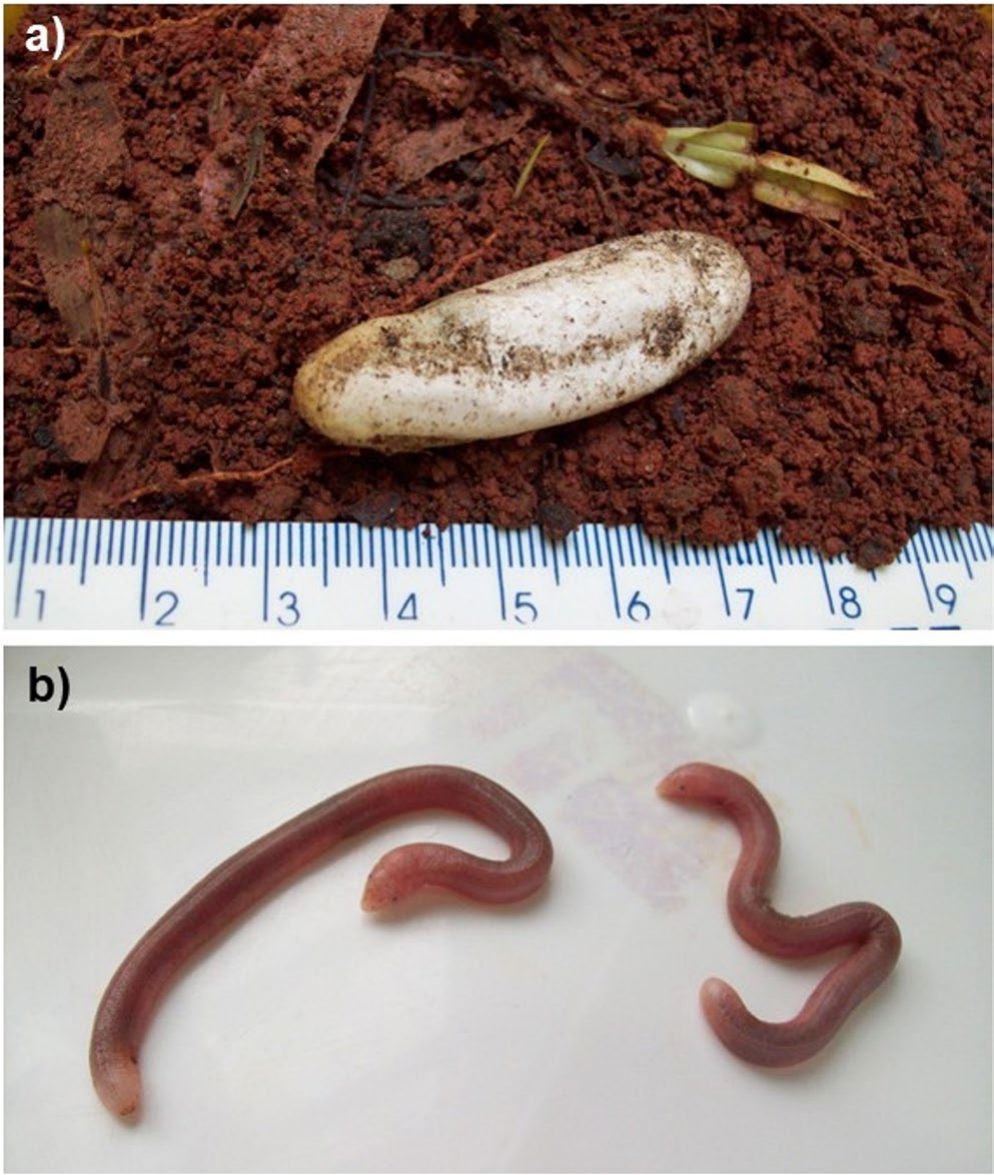
An egg (**a**) and hatchlings (**b**) from a clutch of the smallhead worm lizard, *Leposternon microcephalum*, found in a natural nest in Praia Grande, São Paulo, southeastern Brazil

The clutch of three eggs was found in a cavity 18 cm below the soil surface in a small garden (0.9 × 1.6 m) containing ornamental cacti. The garden was located within a private residence and received direct sunlight through a glass brick wall. The garden was searched for additional eggs and for nearby ant or termite nests but none were found. The eggs had whitish, leathery shells (Fig. 1a). Two eggs hatched within the plastic bag on 27 February 2011, during transport to the laboratory. One hatchling (IBSP.CRIB 295) measured 120 mm snout-vent length (SVL), had a 6 mm tail, and weighed 2.73 g. The other (IBSP.CRIB 296) measured 128 mm SVL, had an 8 mm tail, and weighed 3.52 g. Both hatchlings left substantial unabsorbed residual yolk (1.47 and 1.05 g, respectively) within the eggshells. The remaining egg (45.26 × 15.31 mm, 5.08 g; Fig. 1a) did not hatch. Dissection revealed a full-term dead embryo (133 mm SVL, 9 mm tail length; MZUSP 103179) with residual yolk. All individuals were identified as *L. microcephalum* (Fig. 1b). All 31 excavated ant nests were active, but we found no amphisbaenians and no eggs in any of them. However, we found a blindsnake (*Leptotyphlops* sp.) inside an *Acromyrmex* nest.

### Literature data

Our literature survey identified 18 records of confirmed natural oviposition sites for nine amphisbaenian species from three genera (*Amphisbaena*, *Leposternon*, and *Rhineura*) across South, Central, and North America (Table 1). One additional report described a gravid *A. mertensii* inside a termite nest, suggesting—but not confirming—a potential oviposition site (Table 1). We treated this record separately from confirmed oviposition sites.

Among confirmed records (*n* = 18), oviposition sites were most often in ant nests (*n* = 7, 38.9%) and underground cavities (*n* = 6, 33.3%), followed by sites beneath decaying logs or decomposing wood (*n* = 4, 22.2%) and termite nests (*n* = 1, 5.6%). For most species, records are limited to one or two observations. Multiple records were available only for *L. microcephalum* (*n* = 5) and *A. darwinii* (at least 6, as the original sources do not provide an exact count of distinct oviposition records; Table 1). In *L. microcephalum*, oviposition sites were in ant nests (3/5, 60%) and in underground cavities (2/5, 40%). In *A. darwinii*, half of the records involved underground sites (humus or soil cavities, 3/6), with additional records from beneath a log (at least 2/6) and an ant nest (1/6).

Most records indicated solitary nesting (Table 1). The only unequivocal cases of communal nesting involved *A. darwinii* in Argentina, where groups of 6–8 to 17–21 eggs were occasionally found under the same log. Because *A. darwinii* typically lays 2–4 eggs (Gallardo 1967; Carreira and Baletta 2006), we interpret these records as oviposition by multiple females (~ 2–11). However, solitary oviposition also occurred in this species (Table 1). Two records for *L. microcephalum* were ambiguous regarding oviposition site use. Goeldi (1898) described two sets of eggs differing in shape and developmental stages from a pile of bricks and tiles inhabited by a large *Camponotus* ant colony, which is consistent with more than one clutch. Similarly, Gans (1971) reported two clutches (eight and two eggs) of different sizes and developmental stages from the same locality, but we could not determine whether they originated from a single oviposition site.

## Discussion

### Facultative use of ant and termite nests for egg-laying

Our original observations and literature synthesis do not support the hypothesis that amphisbaenians obligatorily use ant or termite nests for oviposition. Instead, the available evidence indicates that these structures are one option among a broader repertoire of sites. In the two species with multiple records (*L. microcephalum* and *A. darwinii*), eggs have been found in both ant or termite nests and alternative shelters. A similar pattern is observed, though less well documented, in other species. Thus, the evidence supports facultative rather than exclusive use of ant and termite nests, with females exploiting various shelters that meet incubation requirements. Our ant-nest survey, which yielded no amphisbaenians or eggs, is consistent with this conclusion but should be interpreted cautiously given detectability constraints, including sampling on a single date and in human-modified habitats. Importantly, the available evidence addresses dependence (i.e., obligatoriness) rather than preference, because records of eggs outside ant and termite nests indicate that these colonies are not required for oviposition.

While obligate use can be rejected, whether amphisbaenians preferentially select ant and termite nests under certain ecological contexts remains unresolved. The apparent prevalence of these nests in the literature may partly reflect sampling bias, as they are conspicuous, long-lasting, and often searched more intensively than other potential oviposition microhabitats (e.g., soil cavities, burrows, or decaying root systems). Many squamate eggs found in ant and termite nests were discovered incidentally by entomologists during surveys focused on social insects (e.g., Vaz-Ferreira et al. 1970, 1973; Brandão and Vanzolini 1985; Bruner et al. 2012; Kwapich 2021), with dozens to hundreds of nests opened in several cases (e.g., Vaz-Ferreira et al. 1970, 1973; Pramuk and Alamillo 2003). This asymmetry in search effort can inflate the perceived frequency of oviposition in ant and termite nests, even when such sites are used facultatively. Similar patterns of non-exclusive use have been reported for many lizards and snakes (reviewed in Riley et al. 1985; see also Vitt et al. 1997; Khannoon and Evans 2014), suggesting that facultative oviposition in ant and termite nests is a recurrent strategy among squamates. Resolving whether ant and termite nests are used disproportionately relative to their availability will require repeated, standardized surveys across the reproductive season and across multiple potential nesting microhabitats in both natural and human-modified habitats, ideally using transects or grid-based sampling designs. Such surveys should also record microhabitat-level variables, especially soil temperature, soil moisture, nest depth, soil compaction, vegetation cover, colony activity, and brood status.

### Functional hypotheses and trade-offs underlying oviposition in ant and termite nests

Although ant and termite nests are not obligatory oviposition sites for amphisbaenians, they are ecologically relevant options whose use has been recurrently explained by several functional hypotheses. These hypotheses propose that ovipositing within these nests may confer advantages such as buffered microclimates, protection against predators or microbes, and immediate access to food for hatchlings (Hagmann 1907; Kopstein 1928; Vaz-Ferreira et al. 1970; Riley et al. 1985; Hood et al. 2020). However, because these sites are used facultatively, any advantages are expected to be context-dependent and offset by costs, including aggression from resident insects, attraction of predators, constraints on hatchling emergence, and variable antimicrobial environments (Vaz-Ferreira et al. 1970; Oliveira and Della Lucia 1993). Below, we evaluate these benefits and costs in the context of amphisbaenians, emphasizing how trade-offs among them may shape flexible maternal decisions on oviposition-site choice.

#### Microclimatic stability

A frequently proposed explanation for oviposition in ant and termite nests is that they provide buffered and predictable microclimatic conditions that enhance egg survival and offspring performance. These nests can maintain higher and more stable temperature and humidity than adjacent substrates (Vaz-Ferreira et al. 1970; Korb and Linsenmair 2000; Bollazzi and Roces 2002), conditions often assumed to be favorable for squamate eggs (Kopstein 1928; Vaz-Ferreira et al. 1970; Riley et al. 1985; Herrera and Robinson 2000; Velásquez–Múnera et al. 2008; Baer et al. 2009; Rodríguez and Montoya-Lerma 2015; Williams et al. 2022; Sierra-Serrano et al. 2023). This hypothesis is plausible, given the well-established effects of incubation temperature and moisture on offspring traits (While et al. 2018; Bell et al. 2025). However, warmer or more stable conditions do not always improve developmental outcomes, and both the direction and magnitude of effects vary among species. In some cases, less variable incubation temperatures produce hatchlings with phenotypes associated with higher survival than more variable regimes (e.g., Patterson and Blouin-Demers 2008), whereas in others, differences are minimal or absent (Ji et al. 2003; Hao et al. 2006; Li et al. 2013). Unfortunately, no study has directly tested whether amphisbaenian eggs gain fitness-relevant advantages under the microclimatic conditions typical of ant or termite nests, and therefore, the adaptive value of ovipositing in these environments remains uncertain for amphisbaenians. Controlled comparisons between nest-like conditions and alternative shelters are therefore needed to assess whether microclimatic stability favors repeated, but not exclusive, use of these sites.

#### Protection against predators and microbial attack

Another commonly invoked hypothesis is that oviposition within ant and termite nests reduces egg mortality by providing protection against predators and microbial infection. This protection is attributed to both nest architecture and the defensive and hygienic behaviors of resident insects, which may deter egg predators or suppress pathogen growth (Kopstein 1928; Vaz-Ferreira et al. 1970; Riley et al. 1985; Velásquez–Múnera et al. 2008). Although such mechanisms are plausible, empirical support for this hypothesis is inconsistent. Ants have been suggested to clean reptile eggs or embed them in fungus gardens where symbiotic fungi may suppress pathogens (Velásquez–Múnera et al. 2008; Baer et al. 2009); however, high egg mortality has also been documented within social-insect nests. For example, two-thirds of the tegu lizard (*Tupinambis teguixin*) eggs found in a termite nest were rotten (Herrera and Robinson 2000), and mortality has been reported in termite nests for squamates, including amphisbaenians (Beebe 1944; Pramuk and Alamillo 2003). Moreover, resident insects can themselves attack and harm hatchlings and adults (Vaz-Ferreira et al. 1970; Riley et al. 1986; Oliveira and Della Lucia 1993). In addition, ant and termite nests often attract vertebrate predators (e.g., tegu lizards: Beebe 1945; Avila-Pires 1995; snakes: Vaz-Ferreira et al. 1970, 1973; Duleba and Ferreira 2014) that prey on eggs or fossorial squamates (Braz et al. 2014; Kasperoviczus et al. 2015; Rodríguez et al. 2018). Therefore, these observations indicate that any protective role of social-insect nests is context-dependent and cannot be assumed a priori. Testing this hypothesis will require direct comparisons of egg survival and infection rates inside and outside active nests, ideally distinguishing effects of insect behavior or nest architecture from those of underground nesting more generally.

#### Immediate access to food resources

A further hypothesis is that oviposition within ant or termite nests benefits hatchlings by providing immediate access to abundant food resources (Hagmann 1907; Hegh 1922; Hood et al. 2020). This idea is intuitively appealing for insectivorous reptiles, particularly in subterranean environments where prey availability outside nests may be limited. Its relevance, however, depends on whether hatchlings rely on exogenous food shortly after hatching. Squamates show substantial variation in post-hatching feeding strategies. Some hatchling lizards begin feeding within the first 48 h and derive little energy from residual yolk (Radder et al. 2007; Guo et al. 2023), thus rendering immediate food access of limited functional significance. In contrast, other reptiles delay feeding for extended periods and rely largely on residual yolk during early life (Burger 1989; Morafka et al. 2000; Rowe et al. 2017). For amphisbaenians, direct evidence is lacking. Our observations of substantial residual yolk in newly hatched *L. microcephalum* are consistent with limited early dependence on external food sources. Additionally, postnatal residence within ant nest chambers is suggested to be generally brief in squamates (Vaz-Ferreira et al. 1970), limiting opportunities for sustained post-hatching foraging within colonies. These considerations suggest that immediate access to prey is unlikely to be a primary factor influencing oviposition-site choice in amphisbaenians. Targeted data on neonatal feeding behavior and dependence on external food sources are needed to evaluate this hypothesis.

### Can amphisbaenians build their own nests?

It has been assumed that limblessness constrains nest construction in squamates, forcing limbless species to rely primarily on pre-existing structures for oviposition (Packard and Packard 1988; Doody et al. 2009). However, its relevance to specialized burrowing taxa, such as amphisbaenians, remains uncertain. Our record of *L. microcephalum* and historical reports for *A. darwinii* (Berg 1898) document oviposition in subterranean cavities without evidence of ants or termites but do not clarify whether these chambers were excavated by the female or were pre-existing. A more informative account describes at least one clutch of *L. microcephalum* within a smooth, nearly oval underground chamber connected to a distinct access tunnel (Engmann 1927). Although active excavation was deemed possible, the author favored the interpretation that the female modified an existing crevice rather than digging the chamber entirely (Engmann 1927). Subsequent studies of *L. microcephalum* burrowing behavior suggest that both scenarios are mechanically feasible. The species uses its reinforced, shovel-like head and muscular body to penetrate compact soils and maintain tunnel integrity (Gans 1969; Barros-Filho et al. 2008; Hohl et al. 2014), which indicates that it possesses the functional capacity to excavate or substantially modify subterranean spaces, including potential oviposition chambers. These observations challenge the assumption that limblessness precludes nest construction and suggest that some amphisbaenians may actively excavate or modify oviposition chambers, expanding the range of nesting strategies available to fossorial squamates. Direct behavioral observations under controlled conditions will be necessary to investigate nest-site preparation behavior, chamber architecture, and soil manipulation during oviposition.

### Communal oviposition in amphisbaenians

Previous reviews found no evidence of communal oviposition in amphisbaenians (Graves and Duvall 1995; Doody et al. 2009). However, our reexamination of published records indicates that communal oviposition does occur in some South American species, although it appears to be uncommon. Solitary oviposition predominates, yet multiple clutches of *A. darwinii* have been found together in the same microhabitat (Table 1). Communal oviposition in reptiles may arise from social tolerance among females or from aggregation driven by shared responses to limited or favorable nesting sites (Graves and Duvall 1995; Doody et al. 2009). In *A. darwinii*, this interpretation is supported by field observations of adults and juveniles sharing refuges, sometimes in close physical contact (Gallardo 1967; Borteiro et al. 2013). Evidence for communal oviposition *in L. microcephalum* is more equivocal but compatible with occasional aggregation. Therefore, available evidence suggests that communal oviposition occurs in some amphisbaenian species but is neither frequent nor obligate. The coexistence of solitary and communal nesting within amphisbaenians aligns with flexible, context-dependent oviposition strategies. From a behavioral-ecological perspective, communal nesting in amphisbaenians generates testable hypotheses concerning site limitation, microclimatic advantages, and social tolerance, reinforcing the view that oviposition-site choice in this group reflects adaptive plasticity rather than rigid specialization.

## Conclusions

Our original observations and literature synthesis do not support the hypothesis that amphisbaenians obligatorily oviposit in ant or termite nests. The available evidence indicates that these structures are used facultatively among several oviposition-site options that meet their incubation requirements. Although ant and termite nests may offer advantages such as microclimatic buffering or protection, these benefits are likely context-dependent and can be offset by risks including predation, microbial failure, or insect aggression, favoring flexible rather than exclusive use.

Our synthesis also highlights two additional aspects of amphisbaenian reproductive behavior that merit further attention. First, evidence from *A. darwinii* and more strongly from *L. microcephalum* suggests that some amphisbaenians may excavate or modify subterranean chambers for oviposition, challenging the assumption that limbless reptiles rely primarily on pre-existing shelters. Second, records of communal nesting in *A. darwinii*, and possibly in *L. microcephalum*, expand the known behavioral repertoire of amphisbaenians and further support the view that oviposition strategies in this group are variable and context-dependent.

Taken together, these findings indicate that oviposition-site choice in amphisbaenians reflects adaptive plasticity rather than strict specialization or dependence on social insects. Future studies integrating controlled incubation experiments, field surveys across multiple microhabitats, and behavioral observations under natural or semi-natural conditions are necessary to clarify the ecological drivers and adaptive significance of nesting strategies in this group. In particular, combining measures of microhabitat availability with comparable search effort would be especially useful to disentangle preference, availability, and detectability in this system.

## Data Availability

All data supporting the findings of this study are available within the paper.
